# The influence of menstrual cycle phase on neuromuscular performance and subjective perception of effort in elite football players

**DOI:** 10.5114/biolsport.2025.146781

**Published:** 2025-01-20

**Authors:** Blanca Romero-Moraleda, Esther Morencos-Martínez, Patricia Varón, Beatriz Lara, Ester Jiménez-Ormeño, Ana B. Peinado, Jaime González-García

**Affiliations:** 1 Department of Physical Education, Sport and Human Movement, Universidad Autónoma de Madrid, Madrid, Spain; 2 Performance Area, Royal Spanish Football Federation, Las Rozas, 28232 Madrid, Spain; 3 Universidad Francisco de Vitoria, Faculty of Health Sciences, Exercise and Sport Sciences, Pozuelo de Alarcón, Madrid, Spain; 4 Laboratorio de Fisiología del Ejercicio, Facultad HM Hospitales de Ciencias de la Salud de la UCJC, Departamento de Ciencias de la Actividad Física y del Deporte, Universidad Camilo José Cela, C/ Castillo de Alarcón, 49. 28692 Villanueva de la Cañada, Madrid, España; 5 Grupo de investigación en Entrenamiento de Fuerza y Rendimiento Neuromuscular (StrengthP_RG), Facultad HM Hospitales de Ciencias de la Salud de la UCJC, Departamento de Ciencias de la Actividad Física y del Deporte, Universidad Camilo José Cela, C/ Castillo de Alarcón, 49. 28692 Villanueva de la Cañada, Madrid, España; 6 LFE Research Group, Department of Health and Human Performance, Faculty of Physical Ac-tivity and Sport Sciences (INEF), Universidad Politécnica de Madrid (UPM), 28040 Madrid, Spain

**Keywords:** Women, Follicular, Luteal, Resistance, Training, Hormones, VBT, Load

## Abstract

This study aimed to investigate the potential fluctuations in neuromuscular performance and subjective perception of effort during three different phases of the menstrual cycle. Fifteen elite female football players (age: 23.47 ± 6.14) volunteered to participate in the study. Tympanic temperature, saliva hormone measurement, urine concentration of the luteinizing hormone and a calendar tracker were used to verify the following phases of the menstrual cycle: early follicular phase (EFP), late follicular phase (LFP), and mid-luteal phase (MLP). For each phase, mean concentric velocity and the rate of perceived exertion (RPE) with loads that represented 60% and 80% of their 1RM in the half-squat, deadlift and hip thrust exercises were compared. The counter-movement jump (CMJ) test was also performed. Overall, jump height, mean concentric velocity in half-squat, deadlift, and hip thrust exercises with loads at 60% and 80% RM and RPE were all similar in all menstrual cycle phases, without significant differences (p > 0.05). The results of this investigation suggest that vertical jump, mean concentric velocity in three different exercises at different submaximal loads, and RPE for each exercise are not affected in eumenorrheic elite female football players throughout different phases of the menstrual cycle. The study indicated that lower limb mechanical performance in elite female football players remain consistent across menstrual cycle phases. Coaches can confidently monitor and optimize training without undue adjustments related to menstrual cycles.

## INTRODUCTION

Throughout the menstrual cycle, women experience dynamic alterations in the serum concentrations of key female sex steroid hormones. The fluctuations in oestrogen, progesterone, follicle-stimulating hormone, and luteinizing hormone play a pivotal role in regulating ovulatory cycle patterns. These hormonal variations facilitate the classification of the menstrual cycle into two primary phases: the follicular phase and the luteal phase. The follicular phase consists of the early follicular phase (EFP), characterized by low serum concentrations of oestrogen and progesterone, and the late follicular phase (LFP), marked by a peak concentration of oestrogen coinciding with reduced levels of progesterone, concluding with an upswing in luteinizing hormone before ovulation. Subsequently, the luteal phase commences when luteinizing hormone returns to basal levels, featuring elevated concentrations of both oestrogen and progesterone. Peak values of progesterone and high values of oestrogen typically manifest in the middle of the luteal phase (MLP) [1].

Progesterone is acknowledged for its catabolic functions, while oestrogen concentration has been linked to anabolic functionalities and emotional regulation [1–3]. Due to these divergent hormonal functions, speculation arises regarding potential impacts on skeletal muscle adaptations [3] and subjective responses [4, 5] to training between different phases of the menstrual cycle. However, the existing body of literature fails to provide a clear description of this complex interplay [6–12]. Sarwar et al. [10] documented an 11% increase in maximal voluntary isometric force for both quadriceps and handgrip during the ovulation period (estimated at day 14 of the cycle). While these findings suggest a potential association between increased luteinizing hormone and enhanced muscle strength, other studies contradict these results, demonstrating no changes in muscle strength, and/or jumping and force-velocity profiling across different menstrual cycle phases [6–9, 12]. Gür et al. [6] and Janse de Jonge et al. [7] found no differences in concentric and eccentric muscle torque when comparing the menstrual phase, follicular phase, and luteal phase, consistent with the findings of other authors [8, 9]. Specifically, Romero-Moraleda et al. [13] found no variation in velocity and power values across the entire load-velocity profile (i.e., from 20% to 80% of one-repetition maximum – 1RM), or in the estimated 1RM in the squat exercise, suggesting that there are no changes in muscle strength or power values in eumenorrheic women across different phases of the menstrual cycle.

The hormonal fluctuations throughout the menstrual cycle are also involved in the emotional and perceptual responses to exercise [2]. Janse de Jonge et al. [5] highlighted an increased perceived effort during aerobic exercise at high temperatures and humidity in the luteal phase. In contrast, Mattu et al. [4] identified higher perceived effort during a 30-minute exercise at maximal lactate steady state intensity on a cycle ergometer in the follicular phase compared to the mid-luteal phase. These findings on perceived effort in different phases of the menstrual cycle show that the scientific evidence on this matter is inconsistent and very limited – especially in resistance training, where, to the best of our knowledge, no information is available. Therefore, it is necessary to determine whether perceptual differences exist during strength training throughout the various phases of the menstrual cycle.

The contradictory nature of the evidence could be attributed to the use of various methods for estimating menstrual cycle phases [14] and the application of different methods for assessing neuromuscular capacities [10, 13]. A potential limitation of the current evidence is that most studies have measured peak values of muscle strength in isokinetic and isometric muscle contractions, limiting the applicability of the findings to sports training contexts due to the inaccessibility of such evaluations. In this context, the countermovement jump (CMJ) and load-velocity profile emerge as reliable and time-efficient tools for assessing training adaptations and neuromuscular readiness in athletes [15–17]. These not only facilitate daily training decisions based on objective data but also serve as indirect indicators of an athlete’s physical performance, given their association with other markers of sports performance and muscle strength [18, 19]. Moreover, training with submaximal loads guided by the load-velocity relationship has proven to be an effective method to enhance barbell velocity at different submaximal loads [20]. Also, it has demonstrated performance improvements in different sports actions such as vertical and horizontal jumps [21], as well as sprint and change of direction times [22].

Given that CMJ and barbell velocity reflect preparedness to train [16, 17], and this, in turn, can affect long-term neuromuscular adaptations [22, 23], it is essential to identify whether there are variations in jump capacity and barbell velocity due to hormonal fluctuations and emotional regulation changes during different phases of the menstrual cycle. Additionally, identifying variations in these metrics provides insights into neuromuscular function, fatigue levels, and potential imbalances, allowing for tailored adjustments in training programmes. Consequently, this study aimed to explore variations in muscle performance and perceived effort across three menstrual cycle phases (EFP, LFP, and MLP) according to CMJ and barbell concentric velocity at 60% and 80% of 1RM in the half-squat, deadlift, and hip thrust exercises.

## MATERIALS AND METHODS

### Participants

Fifteen elite female soccer players (age: 23.47 ± 6.14 years), belonging to the same team, were recruited for the study. Table 1 displays the descriptive data of the participants. All participants exhibited eumenorrheic cycles (cycles with a duration equal to or greater than 21 days and less than or equal to 35 days, for at least 9 consecutive cycles) and did not use any form of contraceptive hormones in the six months prior to recruitment. The team competed in the Spanish first division during the 2021/2022 season. The players’ menstrual cycle duration was 30.6 ± 3.9 days based on all reported cycles during the season. Participants had 9.4 ± 4.7 years of professional football experience, with a training sessions volume of 5 days per week and one or two official matches during the season. The strength training experience performing 3 to 5 strength sessions per week was at least 3 seasons previously. Furthermore, all participants were free from any type of menstrual disorders (e.g., dysmenorrhoea, amenorrhoea, or heavy symptoms associated with pre-menstrual syndrome), had no musculoskeletal injuries in the three months before the investigation, had at least three seasons of strength training performing 3 to 5 strength sessions per week, and were not taking drugs or dietary supplements during the duration of the experiment. During the experimental period, participants were encouraged to maintain their usual diet and training routines. Two weeks before the onset of the experiment protocol, informed consent was obtained from each participant. The experimental protocol was approved by the Human Ethics Committee of Universidad Autónoma de Madrid (CEI-124-2528), in accordance with the Declaration of Helsinki.

**TABLE 1:** Description of the characteristics of the participants.

		Early Follicular Phase (EFP)	Late follicular phase (LFP)	Mid luteal Phase (MLP)

	Mean ± SD	Mean ± SD	Mean ± SD	Mean ± SD
Age (years)	24.00 ± 3.00	–	–	–
Height (m)	1.68 ± 0.11	–	–	–
BS 1RM (Kg)	73.95 ± 11.53	–	–	–
DL 1RM (Kg)	97.93 ± 18.65	–	–	–
HT 1RM (Kg)	156.06 ± 23.87	–	–	–
Lenght of menstrual cycle (days)	27.00 ± 2.00	–	–	–
Body mass (kg)	58.98 ± 9.15	56.5 ± 5.78	56.42 ± 6.04	56.62 ± 6.36
Tympanic temperature (ºC)	36.41 ± 0.50	36.34 ± 0.42	36.43 ± 0.62	36.42 ± 0.47
Estradiol (pg/mL)	–	5.09 ± 1.44	22.45 ± 17.45	8.43 ± 2.41
Progesterone (pg/mL)	–	33.24 ± 3.91	49.31 ± 5.51	91.22 ± 8.92

SD = standard deviation; 1RM = one repetition maximum; BS = back squat; DL = Deadlift; HT = Hip Thrust

### Study design

**FIG. 1:**
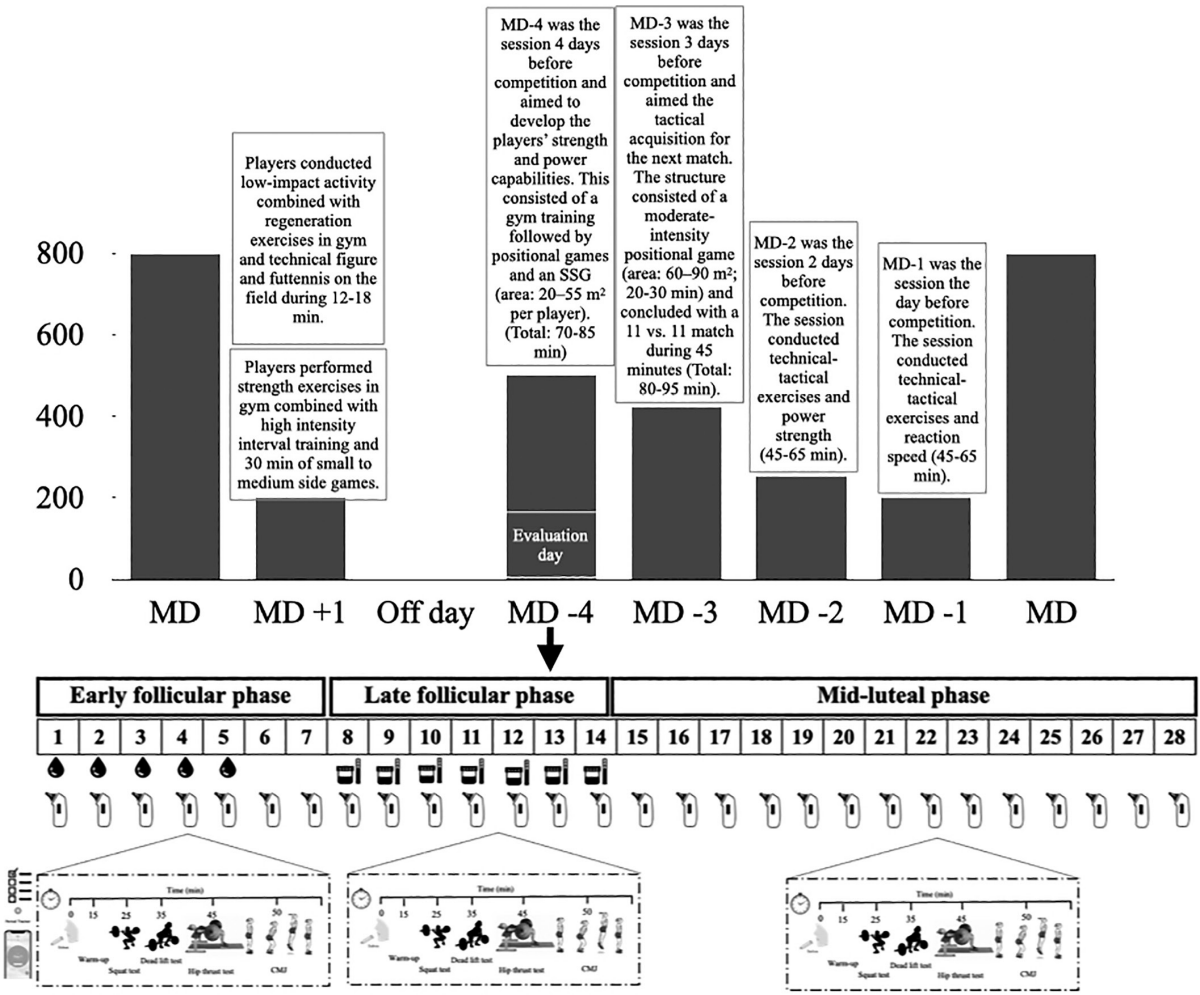
Experimental design of the research

Mean velocity and subjective perception of effort during half-squat (HS), deadlift (DL), and hip thrust (HT) exercises at 60% and 80% of 1RM across three distinct phases of the menstrual cycle were observed. The chosen phases, namely EFP, LFP, and MLP, correspond to significant events in the menstrual cycle. To enhance the ecological validity of the results, the players were assessed on the same day of the week, with the same hours of recovery relative to the last match (Wednesday, Match Day (MD)-4, after 44 hours of rest) at the regular sports facilities where they normally train. To ensure that all female players completed at least one full menstrual cycle in each of its phases, neuromuscular performance was monitored over a period of 5 months. This was confirmed by hormonal analysis, ensuring the accurate identification of each menstrual cycle phase used for statistical analysis. The typical weekly training and competition structure across the study was: MD +1 (i.e. Monday training session, after MD) starter players completed a recovery training session, whereas non-starters completed a compensatory training session. Tuesday was an off-day. The MD-4 (Wednesday) and MD-3 (Thursday) training sessions were focused on strength and conditioning drills (in the gym), plus tactical drills imbedded in small–medium-sided (MD-4) and medium–large-sided (MD-3) soccer pitches. On MD-2 (Friday), skills and strategy exercises and power strength in the gym were the focus. On MD-1 (Saturday), strategy exercises, small side games and velocity training were mainly performed, and the MD was usually scheduled on Sunday. To address the research aim, the participants performed one repetition of the half-squat back, deadlift and hip thrust exercises at maximal intended velocity with loads of 60% and 80% of their respective 1RM, and they were asked to estimate the rate of perceived effort on a 1–10 scale (RPE 1–10). This assessment was performed in each menstrual cycle phase (Figure 1). The testing protocol was randomized for each participant (six started in the EFP, four started in the LFP and the remaining five started in the MLP). The day before each trial, participants were instructed only to rest training and maintain their usual diet/fluid intake routine.

### Experimental procedures

#### Familiarization, 1RM assessment and rate of perceived exertion (RPE)

Two weeks before the commencement of the experiment, participants underwent two familiarization sessions with the testing protocol to mitigate potential learning effects during the study. One week prior to the experiment, a one-repetition maximum (1RM) test for the half-back squat, deadlift, and hip thrust was conducted to standardize the loads for subsequent experimental sessions. The execution order of the three exercises was randomized and consistent across sessions for each of the players. The 1RM assessment involved sets with increasing loads estimated to be approximately 20% of 1RM (3 repetitions), 40% of 1RM (3 repetitions), 60% of 1RM (3 repetitions), 80% of 1RM (1 repetition), and 90% of 1RM (1 repetition).

Participants were then allowed a maximum of five attempts for their 1RM, with incremental load adjustments (0.5 to 2.5 kg) following each successful attempt. The last successful lift with correct technique was recorded as the 1RM load [24]. Rest intervals included two minutes between warm-up sets, five minutes between 1RM attempts, and five minutes between exercises. Subsequently, participants completed three identical experimental trials for each menstrual cycle phase. All testing protocols took place in the morning (between 9 and 11 AM) in a gym setting before the team’s combined gym and pitch session (Figure 1). Upon arrival at the gym, participants provided a saliva sample, which was frozen for later analysis. A standardized 15-minute warm-up protocol was followed, consisting of 5 minutes of cycling on a cycle ergometer (60–90 W), mobility exercises, and bodyweight half-squats, deadlifts, and hip thrusts. For completion of warm-up, participants performed one set of 6 reps with 20% of 1RM for each exercise. After the warm-up, participants performed two reps in the free weight half-squat, deadlift and hip thrust exercises at maximal velocity with loads that represented 60% and 80% of their 1RM. For each load, the RPE was recorded.

Participants were familiarized with the Rating of Perceived Exertion (RPE) scale (1–10) and were asked to provide their RPE after each repetition using a visual scale [25]. Two minutes of passive rest was allocated between the attempts and three minutes between exercises. The full range of motion for the half-back squat involved lowering the body to a 90º knee angle until touching a bench. For the deadlift, participants had to lift the bar without engaging in a countermovement with the hips, concluding the movement in a full-arm-knee-hip extension with the shoulders blocked. A self-selected grip width with a pronated grip was employed. Subsequently, subjects were directed to pull the bar in a vertical direction with maximal intended velocity until their body achieved a full erect position. They were then required to maintain this static position for approximately 1 second. For the hip thrust, participants positioned their upper backs on a bench, spaced their feet slightly wider than shoulder-width apart, and pointed their toes forward or slightly outward. A thick bar pad was used to cushion the barbell, which was positioned over the subjects’ hips. They were then instructed to thrust the bar upward while ensuring a neutral spine and pelvis. Execution technique and motivation were standardized and monitored by two experienced researchers for subject safety and experimental condition reliability. Barbell mean velocity (in m/s) during the concentric phase was determined using a linear encoder (CLTP, Chronojump, Boscosystem, Barcelona, Spain), and the attempt with the highest mean velocity at each load was used for statistical analysis.

### Countermovement jump assessment

Countermovement jump (CMJ) height was used to determine low-force-high-velocity measurements. All participants performed two maximal CMJ attempts (separated by 1 minute) with arms akimbo. Subjects were requested to maintain their hands on their hips during the jump. Knee flexion was not allowed during the flight phase. If any of these parameters were not followed, the trial was repeated. All the attempts were performed on a Force-Decks FD4000 Dual Force platform (ForceDecks, London, United Kingdom) with a sampling rate of 1,000 Hz. Centre-of-mass (COM) velocity was calculated by dividing the vertical force (subtracting body weight) by body mass and integrating the product using the trapezoid rule. Instantaneous power was determined by multiplying the vertical force by the COM velocity. COM displacement was obtained through double integration of the vertical force data [26]. A CMJ was considered successful if performed with the arms akimbo, and participants maintained complete stillness for at least one second during the weighing phase [27]. The initiation of the movement was identified when a drop of 20 N from baseline force (recorded during the weighing phase) was observed. The concentric phase started when velocity became positive and ended at take-off. In accordance with the conceptual framework developed by Bishop et al. [28], we selected the following variables from the CMJ due to their appropriate reliability [29] and their reflection of different aspects of vertical jump performance (i.e., outcome, kinetics, kinematics, and jump strategy) [27]. Jump metrics were defined as follows: jump height (the highest displacement of the centre of mass calculated from vertical velocity at take-off), concentric mean power relative to body mass (BM) (mean value of instant power during the concentric phase), concentric mean force relative to BM (mean value of instant force during the concentric phase) and concentric duration (the time, in ms, from the onset of positive velocity to take-off).

### Determination of the menstrual phase and hormonal analysis

The accurate determination of menstrual cycle monitoring and the identification of each phase followed previous recommendations [14, 30]: (a) verification of sex hormones with saliva samples, (b) period tracker application five months previously, and (c) assessment of the urinary peak of luteinizing hormone, which was conducted to confirm the menstrual cycle phase and exclude deficient luteal phases.

Saliva samples were collected in each menstrual cycle phase for measuring oestradiol (17β-oestradiol; E2) and progesterone (P) between the 2^nd^ and the 5^th^ day (mean ± SD: 3.23 ± 1.30 day) of the cycle for the EFP, between one and three days before the ovulation day (mean ± SD: 10.84 ± 1.91 days) for LFP and between five and nine days following ovulation (mean ± SD: 20.58 ± 2.11 days) for MLP. Saliva was collected using the Salimetrics (CA, USA) salivary Estradiol and Progesterone Immunoassay Kits. Players were instructed to use straws until at least 50% of the tube was filled. Afterwards, samples were placed in a freezer at -20ºC. E2 and P-samples were analysed using enzyme-linked-immunosorbent assay (ELISA) kits (IBL, Hamburg, Germany), which are all validated by LC-MS according to the manufacturer. All samples were analysed in one run with assay kits from the same lot to reduce inter-assay variability. Moreover, all samples were analysed in duplicate. The mean intra-assay coefficient of variation across the whole range of standards was 12.51% for E2 and 23.82% for P. Sex hormone concentrations (E2 and P) throughout the menstrual cycle phases are shown in Table 1.

The tracker period was performed using a mobile application (Mycalendar, Period-tracker, US) in conjunction with a menstruation diary containing details such as the date of menses, length of menses, and discomfort in the days preceding and during menses. Additionally, participants were provided with 7 reactive test strips (One Step Ovulation LH Test Strip; CVS Corporation, US) to assess any increase in luteinizing hormone in the first-morning urine sample 1 and 3 days preceding expected ovulation. Using this information, the onset of the EFP was indicated by the initiation of menses, and the testing protocol was conducted between 1 and 6 days. The LFP was confirmed by a positive test for urinary luteinizing hormone, and the testing protocol was implemented between days 11 to 15. The MLP was determined to be between 70% and 75% of the individual menstrual cycle length (i.e., from the 19^th^ to the 24^th^ day of the menstrual cycle for a regular cycle of 28 days) [30, 31]. All these protocols were instrumental in aligning the participants’ menstrual cycles with the temporal windows for each phase, accommodating different cycle lengths and the constraint of having only one day per week to execute the testing protocol. Participants who did not align with their designated phase on the testing protocol day underwent testing in the subsequent menstrual cycle.

### Statistical analysis

Statistical analyses were performed utilizing IBM SPSS Statistics version 26.0 (IBM Corp., Armonk, NY, USA). The data obtained during the familiarization displayed a normal distribution after the Shapiro-Wilk test. In cases where the assumption of sphericity was not met, the Greenhouse-Geisser sphericity correction was applied. The standard error of measurement (SEM) and coefficient of variation (CV%) were calculated to identify absolute reliability. The criterion for acceptable reliability was set at CV ≤ 10% [32]. The differences among the phases of the menstrual cycle were analysed using oneway repeated-measures analysis of variance (ANOVA). The statistical threshold was set at *p* ≤ 0.05. Bonferroni correction was applied in each pairwise comparison. Additionally, effect sizes (ES) with a ± 90% confidence interval (CI) were used on log-transformed data to mitigate bias arising from the non-uniformity of error. ES were interpreted based on the following ranges: < 0.2, trivial; 0.2–0.6, small; 0.6–1.2, moderate; 1.2–2.0, large; 2.0–4.0, very large; and > 4.0, extremely large [33].

## RESULTS

All mean concentric velocities (V^mean^HS60: SEM = 0.05 m/s, CV = 5.28%; V^mean^HS80: SEM = 0.05 m/s, CV = 7.41%; V^mean^DL60: SEM = 0.05 m/s, CV = 7.26%; V^mean^DL80: SEM = 0.05 m/s, CV = 8.59%; V^mean^HT60: SEM = 0.04 m/s, CV = 6.45%; V^mean^HT80: SEM = 0.04 m/s, CV = 7.21%) and CMJ metrics meet the acceptable reliability threshold (jump height: SEM = 0.99 cm, CV = 3.06%; mean concentric power/kg: SEM = 1.09 W/kg, CV = 3.16%; mean concentric force/kg: SEM = 0.46 N/kg, CV = 1.66%; concentric duration: SEM = 12.25 ms, CV = 3.86%).

**FIG. 2:**
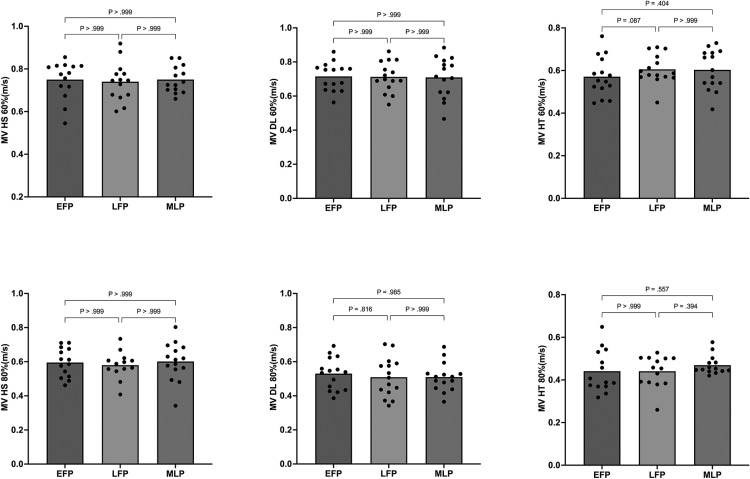
Mean (bars) and individual responses (dots) of the average concentric velocity in the HS, DL, and HT exercises at 60% and 80% of 1RM during each phase of the menstrual cycle.

There was no main effect of the menstrual cycle phase on any of the analysed concentric mean velocities – HS60%RM (F^2**,**28^ = 0.147, p = 0.864; η^2^ = 0.011), HS80%RM (F^2**,**28^ = 0.130, p = 0.878; η^2^ = 0.009), DL60%RM (F^2**,**28^ = 0.028, p = 0.973; η^2^ = 0.002), DL80%RM (F^2**,**28^ = 0.680, p = 0.515; η^2^ = 0.046), HT60%RM (F^2**,**28^ = 2.458, p = 0.104; η^2^ = 0.149), or HT80%RM (F^2**,**28^ = 1.560, p = 0.229; η^2^ = 0.107). Mean and individual values are displayed in Figure 2.

No main effect of the menstrual cycle was observed in any of the vertical jump metrics. Jump height (F^2**,**28^ = 0.829; p = 0.453; η^2^ = 0.084), mean concentric power/BM (F^2**,**28^ = 3.049; p = 0.085; η^2^ = 0.253), and mean concentric force/BM (F^2**,**28^ = 3.168; p = 0.089; η^2^ = 0.260) were similar across the three phases of the menstrual cycle analysed (Table 2). Additionally, the jump strategy was consistent across the three menstrual cycle phases, with no significant main effect observed for the duration of the concentric phase (F^2**,**28^ = 2.795; p = 0.115; η^2^ = 0.237). The weight of the participants was similar across all phases of the menstrual cycle (F^2**,**28^ = 0.463; p = 0.637; η^2^ = 0.0.049).

**TABLE 2:** Differences in the CMJ metrics throughout the different phases of the menstrual cycle

	Early Follicular Phase (EFP)			Late Follicular Phase (LFP)			Mid Luteal Phase (MLP)		

	Mean ± SD	P-value (EFP VS LFP)	ES (90%CI)	Mean ± SD	P-value (LFP VS MLP)	ES (90%CI)	Mean ± SD	P-value (EFP VS MLF)	ES (90%CI)
JH (cm)	30.77 ± 4.38	> 0.99	-0.02 (-0.20 to 0.16)	30.79 ± 3.75	> 0.99	0.17 (-0.09 to 0.44)	31.37 ± 3.52	0.828	0.14 (-0.10 to 0.38)

ConPow/BM (W/Kg)	25.74 ± 3.20	> 0.99	-0.07 (-0.35 to 0.22)	25.30 ± 2.90	0.175	0.55 (0.13 to 0.96)	26.90 ± 3.08	0.465	0.33 (-0.06 to 0.72)

ConForce/BM (N/Kg)	19.03 ± 1.47	> 0.99	-0.03 (-0.32 to 0.25)	18.84 ± 1.39	0.121	0.49 (0.12 to 0.87)	19.61 ± 1.92	0.536	0.35 (-0.08 to 0.77)

Concentric duration (ms)	270.00 ± 39.28	0.89	0.05 (-0.25 to 0.34)	276.00 ± 38.60	0.056	-0.40 (-0.75 to -0.04)	259.80 ± 47.14	0.873	-0.28 (-0.71 to 0.15)

JH = jump height; ConPow = conentric power; ConForce = Conentric force; BM = Body mass; ES = effect size; CI = Confidence interval; ms = miliseconds

Table 3 presents the results of RPE in different phases of the menstrual cycle. There were no significant differences in RPE for either of the two loads (i.e. 60% and 80% of 1RM) in any of the three evaluated exercises, all p-values being > 0.99 and ES lower than 0.39.

**TABLE 3:** Differences in the rate of perceived effort (RPE) throughout the different phases of the menstrual cycle.

		Early Follicular Phase (EFP)			Late Follicular Phase (LFP)			Mid Luteal Phase (MLP)		

		Mean ± SD	P-value (EFP VS LFP)	ES (90%CI)	Mean ± SD	P-value (LFP VS MLP)	ES (90%CI)	Mean ± SD	P-value (EFP VS MLF)	ES (90%CI)
**RPE(AU)**	BS 60% 1RM	5.33 ± 1.11	> 0.99	-0.21 (-0.61 to 0.18)	5.13 ± 0.34	> 0.99	0.14 (-0.24 to 0.53)	5.26 ± 0.26	> 0.99	-0.03 (-0.45 to 0.38)

	BS 80% 1RM	7.40 ± 0.81	> 0.99	0.12 (-0.30 to 0.54)	7.50 ± 0.78	> 0.99	0.02 (-0.46 to 0.50)	7.53 ± 0.92	> 0.99	0.14 (-0.30 to 0.58)

	DL 60% 1RM	5.33 ± 0.90	> 0.99	0.39 (-0.10 to 0.89)	5.83 ± 1.38	> 0.99	-0.21 (-0.66 to 0.25)	5.47 ± 1.19	> 0.99	0.08 (-0.42 to 0.58)

	DL 80% 1RM	7.77 ± 1.07	> 0.99	0.15 (-0.21 to 0.51)	7.93 ± 1.03	> 0.99	0.07 (-0.46 to 0.60)	8.00 ± 0.93	> 0.99	0.22 (-0.18 to 0.62)

	HT 60% 1RM	6.20 ± 1.08	> 0.99	-0.05 (-0.41 to 0.32)	6.13 ± 1.13	> 0.99	0.14 (-0.27 to 0.54)	6.23 ± 0.68	> 0.99	0.08 (-0.20 to 0.36)

	HT 80% 1RM	8.57 ± 1.07	> 0.99	-0.26 (-0.68 to 0.16)	8.27 ± 0.88	> 0.99	-0.01 (-0.43 to 0.40)	8.23 ± 0.68	> 0.99	-0.27 (-0.56 to 0.02)

BS = back squat; RM = repetition maximum; DL = deadlift; HT = hip thrust; SD = standard deviation

## DISCUSSION

This research had two main objectives: i) to explore potential variations in neuromuscular performance by measuring various metrics derived from the countermovement jump (CMJ) and mean concentric velocity in half-squat, deadlift, and hip thrust exercises with loads at 60% and 80% of 1RM; and ii) to identify whether differences in perceived exertion existed across EFP, LFP, and MLP phases of the menstrual cycle. Notably, this study is among the few to investigate the effects of the menstrual cycle on both neuromuscular performance and the perception of exertion in elite female football players, an area that remains underexplored in current sports science research. In summary, the results of this study showed that there were no changes in vertical jump performance or mean concentric velocity in the analysed exercises and loads. The lack of variation throughout the menstrual cycle is also reflected in RPE values, as no significant differences were observed between menstrual cycle phases. These findings suggest that hormonal fluctuations during the menstrual cycle do not affect either neuromuscular performance or perceived effort in elite female football players.

The CMJ reflects the muscular capacity to generate maximum force in a specific short period, as well as to produce force at elevated contractile velocity during stretch-shortening cycle (SSC) movements. Therefore, it represents one of the representative expressions of explosive lower-body power [34] and demonstrates a clear association with muscular strength [19], emphasizing its relevance for athletic performance. Despite theoretical implications suggesting that hormonal fluctuations corresponding to each phase of the menstrual cycle may affect the production of force and muscular power in explosive SSC actions, the literature on this topic is limited [12]. The concentration of steroid hormones can affect the laxity of tendons and ligaments, leading to modifications in musculotendinous stiffness. Therefore, theoretically, vertical jump performance may be diminished during the ovulation phase compared to the follicular and menstrual phases [35]. However, the results obtained in the present study do not support this idea. Our findings suggest that jumping performance is not affected by the different menstrual cycle phases analysed, as demonstrated by the non-significant and trivial or small ES differences observed in jump height, concentric power and force, and concentric duration (Table 2). However, to the best of our knowledge, this is the only study that has analysed the kinetics, kinematics, and strategy of vertical jumps across different phases of the menstrual cycle, making it challenging to compare our results. In a study involving professional female soccer players, comparable results for jump height were observed, with the CMJ height showing no significant difference between the EFP and MLP conditions (p = 0.33; ES = 0.16; trivial) [36]. Furthermore, the force and power data presented in this research are relative to body weight. Therefore, these results could be biased by potential weight gain due to fluid retention during the luteal phase of the menstrual cycle [37]. However, the body weight of the athletes in this study remained similar across all phases. It seems that fluctuations in oestrogen and progesterone concentrations throughout the different phases of the menstrual cycle do not modify force production in an explosive SSC action such as the CMJ.

Neuromuscular adaptations following strength training tend to be velocity-specific in resistance trained populations [23, 34], showing distinct adaptations in different areas of the load-velocity profile [18, 20]. Therefore, if force (and velocity) production were limited during any phase of the menstrual cycle, it could result in lower neuromuscular adaptations in the long term, or vice versa [38]. Notably, transcranial magnetic stimulation studies indicate that during the EFP, when oestradiol concentration peaks, there could be greater cortical excitation, which may lead to higher force and velocity production. In contrast, the phases of the EFP and MLP are characterized by cortical inhibition that might hinder force production during the contractile process [39, 40]. Also, it is possible that oestrogen-induced inhibition of the neurotransmitter gamma-aminobutyric acid A (GABA) could enhance performance during ovulation [39, 40]. Despite this context, our results do not indicate any menstrual cycle effect on a moderate load (oriented towards power production) or a high load (oriented towards force production at lower contractile velocities) in three basic resistance exercises. Our results align with the conclusions of prior studies [6, 8, 9, 41, 42] where researchers found no variations in peak force production across menstrual cycle phases. However, it is noteworthy that the muscle strength tests commonly employed in earlier studies involved the handgrip test and isokinetic testing for knee flexors and extensors. While these tests are reliable for assessing maximal force values, they do not provide information about force production during submaximal loads. In this regard, we have only identified one study that has analysed movement velocity across different loads in the loadvelocity profile [13]. According to our results, Romero-Moraleda et al. [13] did not observe any difference in movement velocity across the entire load-velocity profile (i.e. 20 to 80%RM) in the guided halfsquat exercise. Although our findings and previous studies indicate that there are no acute differences in force and velocity production across different phases of the menstrual cycle, this does not contradict the idea that hormonal fluctuations could lead to differences in other key adaptations after strength training, such as increased strength and muscle hypertrophy. Oestrogen participates in various signalling pathways in skeletal muscle, including 5’AMP-activated protein kinase (AMPK), which regulates transcription. Additionally, oestrogen activates satellite cells and inhibits protein catabolism, the opposite of progesterone’s action [3]. Consequently, there is a greater potential for recovery and reconstruction of muscle fibres after exercise during the mid-follicular phase [3]. Therefore, resistance training during the follicular phase may induce a more robust muscular anabolic response than during the luteal phase, due to elevated oestrogen levels and low progesterone levels in the follicular phase [3, 43].

While neuromuscular responses throughout the menstrual cycle have been extensively researched, the perceptual response to strength exercises lacks existing studies despite its practical implications. For coaches and fitness trainers, it is essential to identify and monitor individual responses of athletes to training loads to design an appropriate training cycle [44]. In this regard, understanding whether the exercise response varies based on the menstrual cycle is important. A recent review [2] on perceptual responses during the menstrual cycle suggested that during ovulation, there is higher motivation and competitiveness compared to the luteal and/or follicular phases. Additionally, negative sensations are reported during the premenstrual phase (i.e., mood disturbances). These emotional differences could be explained by higher testosterone concentrations during ovulation, as this hormone has been shown to be related to motivation to perform [45], which could modify the perceptual response to exercise due to a different motivational state. However, the findings of our research, the first to identify the perceived effort in different resistance exercises at different loads, suggest that there is no difference in RPE values between the analysed phases of the menstrual cycle (Table 3). Despite the differences in the type of tasks performed, Paludo et al. [2] also observed that there was no variation in RPE values after exercise in the different phases of the menstrual cycle. However, it is necessary to interpret our results with caution, as the perceived effort of the present research corresponds to a minimal training volume (indeed, the only training volume is that of the assessments themselves). Therefore, our results should not be generalized to other tasks of greater duration and intensity.

This study has several limitations that should be acknowledged and discussed. Firstly, this study has been performed in ecological conditions. Due to the nature of the research, numerous evaluations were required across different cycles to obtain a complete cycle that aligned with the measurement days and the established phase definition criteria. Additionally, the state of wellbeing of the female soccer players, which could influence their performance, was not considered [46]. These limitations should be addressed in future research, particularly in studies exploring how the state of wellbeing and the different phases of the menstrual cycle influence neuromuscular performance in female athletes during strength and endurance tests.

It is also important to note that the sample is not representative of the general population but rather consists of elite athletes, limiting the extrapolation of the results to the general population.

## CONCLUSIONS

Our findings suggest that vertical jump, mean concentric velocity in three different strength exercises and across various zones of the load-velocity profile, and RPE for each exercise, are not affected in eumenorrheic elite women football players throughout different phases of the menstrual cycle. Coaches and fitness trainers can use these findings to understand the acute mechanical response and feel more confident when monitoring performance, avoiding unnecessary adjustments and overanalysis related to menstrual cycle phases.
